# Using a reflective practice group to facilitate the professional development of care staff in a dementia care setting

**DOI:** 10.1192/j.eurpsy.2025.1758

**Published:** 2025-08-26

**Authors:** L. L. Tan, H. K. Wong

**Affiliations:** 1 Psychological Medicine, Changi General Hospital, Singapore, Singapore

## Abstract

**Introduction:**

Research has shown that staff working in clinical environments caring for frail, elderly or dying patients often must deal with high levels of emotional distress and psychological pain. Psychological defences are necessary and are part of our normal coping mechanism to deal with grief and losses but if they are not understood and recognized, may impact on staff and organizations in unhelpful and destructive ways. In resilient caregiving organizations, emotions are respected and attending to these emotions allow staff to create relationships which will help them cope with their work better.

**Objectives:**

Through interprofessional education with a reflective group, it was hoped that the improved understanding of emotions and experiences of staff and patients could directly influence clinical practice.

**Methods:**

Participants were invited to monthly meetings of 60 minutes for 12 months. A psychodynamic perspective addressed the unconscious processes in clinical encounters. Participants were encouraged to describe how they felt and the meanings of their behaviour rather than just focusing on what happened. Group size was capped at 12 with 2 psychodynamically-oriented facilitators. Semi-structured interviews were conducted and a qualitative approach with content analysis of the transcribed interviews was adopted.

**Results:**

Interdisciplinary staff included nurses, psychologists, social workers, junior and senior clinicians. Our findings showed that staff in dementia care encountered significant levels of emotional distress. The major themes emerged included:

1. Universality of emotions
2. Psychologically safe space
3. Enhancement of reflective capacity
4. Sense-making in the clinical environment

**Conclusions:**

The experiential group discussions allowed staff to better understand and recognize their vulnerability by providing a safe space for directed catharsis. The enhancement of reflective capacity through mirroring and universality in groups allowed members to create relationships which helped them cope with their work better. Through critical inquiry and dialogue, there was better awareness of the social, cultural, economic and political forces at work as staff were encouraged to think and respond honestly to day to day clinical and organizational pressures.

**Disclosure of Interest:**

None Declared

